# Melanopsin and Cone Photoreceptor Inputs to the Afferent Pupil Light Response

**DOI:** 10.3389/fneur.2019.00529

**Published:** 2019-05-22

**Authors:** Andrew J. Zele, Prakash Adhikari, Dingcai Cao, Beatrix Feigl

**Affiliations:** ^1^Institute of Health and Biomedical Innovation, Queensland University of Technology (QUT), Brisbane, QLD, Australia; ^2^School of Optometry and Vision Science, Queensland University of Technology (QUT), Brisbane, QLD, Australia; ^3^Department of Ophthalmology and Visual Sciences, University of Illinois at Chicago, Chicago, IL, United States; ^4^School of Biomedical Sciences, Queensland University of Technology (QUT), Brisbane, QLD, Australia; ^5^Queensland Eye Institute, Brisbane, QLD, Australia

**Keywords:** pupil light reflex, melanopsin, cone, photoreceptor, intrinsically photosensitive retinal ganglion cells

## Abstract

**Background:** Retinal photoreceptors provide the main stage in the mammalian eye for regulating the retinal illumination through changes in pupil diameter, with a small population of melanopsin-expressing intrinsically photosensitive retinal ganglion cells (ipRGCs) forming the primary afferent pathway for this response. The purpose of this study is to determine how melanopsin interacts with the three cone photoreceptor classes in the human eye to modulate the light-adapted pupil response.

**Methods:** We investigated the independent and combined contributions of the inner and outer retinal photoreceptor inputs to the afferent pupil pathway in participants with trichromatic color vision using a method to independently control the excitations of ipRGCs, cones and rods in the retina.

**Results:** We show that melanopsin-directed stimuli cause a transient pupil constriction generated by cones in the shadow of retinal blood vessels; desensitizing these penumbral cone signals uncovers a signature melanopsin pupil response that includes a longer latency (292 ms) and slower time (4.1x) and velocity (7.7x) to constriction than for cone-directed stimuli, and which remains sustained post-stimulus offset. Compared to melanopsin-mediated pupil responses, the cone photoreceptor-initiated pupil responses are more transient with faster constriction latencies, higher velocities and a secondary constriction at light offset. The combined pupil responses reveal that melanopsin signals are additive with the cone signals.

**Conclusions:** The visual system uses the L–, M–, and S–cone photoreceptor inputs to the afferent pupil pathway to accomplish the tonic modulations of pupil size to changes in image contrast. The inner retinal melanopsin-expressing ipRGCs mediate the longer-term, sustained pupil constriction to set the light-adapted pupil diameter during extended light exposures.

## Introduction

In humans and non-human primates, melanopsin-expressing ipRGCs have an intrinsic photoresponse (1, 2), receive extrinsic rod and cone inputs and project to the olivary pretectal nucleus (OPN) (1, 3) to form the primary afferent pupil pathway and regulate the pupil aperture (2, 4–12). Pupil diameter is critical for modulating retinal illumination, enhancing visual performance by varying ocular aberrations and depth of focus (13) and is a clinically significant biomarker in neuro-ophthalmology (14, 15). The relative rod, cone and melanopsin-expressing intrinsically photosensitive retinal ganglion cell (ipRGC) contributions to the pupil light response (PLR) have been explored in both animals and humans having different photoreceptor spectral responses and post-receptoral pathways, using different methodological approaches. When all ocular photoreceptors (rods, cones, and ipRGCs) are knocked-out in transgenic mice, there is no PLR (16). In transgenic mice with ipRGCs that do not express the melanopsin photopigment, the PLR is normal at low irradiances and reduced at high irradiances, indicating that rods and cones can contribute to the PLR without activating melanopsin (17, 18). In rod-cone knock-out mice, the PLR is present, but with reduced response amplitude, indicating that melanopsin-expressing ipRGCs alone can mediate the pupil response (17). Similarly, in non-human primates (macaque) following pharmacological blockade of rod and cone signals, the PLR is present with lower amplitude, slower dynamics, and persists after light offset (2); immunotoxin ablation of the OPN4 melanopsin gene in rhesus monkeys results in a reduction in the maximum pupil constriction amplitude and elimination of the post-illumination pupil response (19). When mouse ipRGCs are selectively ablated however, the PLR is absent, indicating the rod-cone pathway does require ipRGCs for a functional pupil response (20). The animal models therefore show that ipRGCs, rods and cones are complementary in their signaling to the pupil control pathway (18, 21–23). However, transgenic animal models that by design, knock-out photoreceptors, cannot be used to independently control the level of activation and interaction between the different photoreceptor inputs to the PLR and so alternate methods are required.

The relative photoreceptor contributions to human PLR can be studied using psychophysical methodologies that independently control the photoreceptor excitations. Outer retinal receptors drive the transient pupil constriction (2, 4, 7, 24–29), but the melanopsin, L–, M–, and S–cone inputs have not been separated in normally-sighted people to identify their independent and combined contributions. After light offset, the redilation of the post-illumination pupil response (PIPR) in the dark is modulated by both rhodopsin and melanopsin during its early-redilation phase (4) and then entirely by melanopsin (2, 30); there has been no direct measurement of the melanopsin control of the PIPR under light-adapted photopic conditions, nor the melanopsin interaction with cone signals. Extrinsic cone inputs to the OPN are mediated via ipRGCs through retinal interneurons (19, 31, 32) and there is evidence for an independent post-retinal pathway for chromatically opponent inputs to the afferent pupil response (26, 33). To determine the melanopsin contribution to the light-adapted PLR, the intrinsic melanopsin response must be separated from the outer retinal (rod and cone) photoreceptor responses. Here we isolate the melanopsin and cone contributions to the PLR for photoreceptor-directed incremental light pulses using a method of silent-substitution (6, 34) that independently controls their relative activity under conditions that provide constant rod photoreceptor excitation. The outcomes of this study reveal the separate and combined contributions of melanopsin and cones to light-adapted, photopic pupil responses in humans with trichromatic color vision.

## Materials and Methods

### Participants and Ethics Statement

All experimental protocols were approved by the Queensland University of Technology (QUT) Human Research Ethics Committee (approval no: 1700000510) and conducted in accordance with their guidelines. Test protocols were completed in compliance with the tenets of the Declaration of Helsinki and all participants provided informed and written consent after the nature and possible consequences of the experiments were explained. Four healthy participants with trichromatic color vision (2 females, 2 males, 23–41 years; one observer was an Author) and no systemic disease took part in this study in accordance with the human research ethics approval. All observers underwent a comprehensive ophthalmic examination, including fundus examination, ocular coherence tomography, color vision (D-15 and Rayleigh color match), visual acuity, contrast sensitivity (Pelli-Robson) and intra-ocular pressure to exclude any retinal or optic nerve disease.

### Apparatus and Calibrations

A calibrated five-primary Maxwellian-view photostimulator with 12-bit resolution and a ~488 Hz upper frequency limit (6) was used to generate all test stimuli. This photostimulator includes five narrowband primary lights comprising light emitting diodes (LED) and interference filters with peak wavelengths (full widths at half maximum) at 456 nm (10 nm), 488 nm (11 nm), 540 nm (10 nm), 594 nm (14 nm), and 633 nm (15 nm) that were combined using fiber optic cables and a homogenizer and focused by an achromatic doublet field lens in the plane of a 2 mm artificial pupil in Maxwellian view. The outputs of the primary lights were controlled by an Arduino based stimulation system, a LED driver (TLC5940), a microcontroller (Arduino Uno SMDR3, Model A000073) and calibrated neutral density filters (Ealing, Natick, MA, USA) using custom engineered software (Xcode 3.2.3, 64-bit, Apple, Inc., Cupertino, CA, USA). The spectral outputs of five primary lights were measured with a spectroradiometer (StellarNet, Tampa, FL, USA); luminance outputs measured with an ILT1700 Research Radiometer (International Light Technologies, Inc., Peabody, MA, USA) as a function of the duty cycle of the LED driver were used to compute the linearization coefficients (6).

The excitations of melanopsin, rhodopsin and the three cone opsins were independently controlled using the principle of silent substitution (6, 34). The L– M– and S–cone, rod (R) and ipRGC (i) excitations were calculated based on CIE 1964 10° standard observer cone fundamentals (35), the CIE 1951 scotopic luminosity function, and melanopsin spectral sensitivity function (30, 36), respectively. For a 1 photopic Troland (Td) light metameric to an equal energy spectrum, the photoreceptor excitation relative to photopic luminance with a 2:1 L:M cone ratio is *l* = L/(L+M) = 0.6667, *m* = M/(L+M) = 0.3333, *s* = S/(L+M) = 1, *r* = R/(L+M) = 1 and intrinsic melanopsin *i* = I/(L + M) = 1. Measurements were performed with a 2000 photopic Td adapting stimulus field chromaticity that had an orange appearance (*l* = 0.752, *s* = 0.105, *r* = 0.319, and *i* = 0.235). Using the principle of silent substitution to selectively modulate one photoreceptor class, or a combination of up to four photoreceptor classes, unique scaling coefficients for the each of the 5-primary lights are calculated using linear algebra (6, 37, 38) for the nominated Weber contrast [C = (Td_max_–Td_min_)/${\text{Td}}_{\text{mi}{\text{n}}^{*}}$100%] of the photoreceptor excitation(s). For example, a 6% Weber contrast +L–M stimulus increases the L–cone excitation by 6% contrast relative to the photoreceptor excitation at the adapting background level, and decreases the M–cone excitation by −6% contrast; the result of this +L–M photoreceptor excitation is a chromaticity change (i.e., a magenta appearing light modulation) without altering the mean retinal illumination or the intrinsic melanopsin-ipRGC, rod and S–cone photoreceptor excitations relative to the adapting background level.

To nullify individual differences in pre-receptoral filtering and photoreceptor spectral sensitivities between the observer and the CIE 1964 10° standard observer, participants performed heterochromatic flicker photometry (HFP) settings between a reference primary (cyan; 100 Td mean illuminance, 15 Hz square wave counterphase flicker) and each of the test primaries (red, green, blue and amber) (38). The 15 Hz modulation frequency is beyond the temporal resolution of the chromatic mechanisms (39, 40), of melanopsin photoreception (10) and therefore likely mediated by the inferred luminance pathway (35, 41). During each HFP setting, the observer minimized the appearance of flicker by controlling the radiance of the test primary using a method of adjustment. For each test-reference wavelength combination, the final setting was the average of 15 repeats; the theoretical 10° standard observer data was then scaled by the observer's average HFP settings.

### Experimental Design: Pupil Light Responses

The stimulus was a 30° diameter circular field with the central 10.5° blocked to eliminate the effect of macular pigment. A small hole (<1 min arc) in the center of this macular blocker was used for fixation. Prior to all experimental sessions, the observers were adapted to the dark-room illumination (< 0.0003 lux) for 15 min followed by a 2 min adaptation to the 2000 Td orange field. In order to maintain a constant retinal illumination during the stimulus presentation (42), consensual pupil responses in the un-stimulated eye were infrared LED illuminated (λmax = 851 nm) and imaged with a camera (640 X 480 pixels; 60 Hz; Point Gray FMVU-03MTM-CS; Richmond, BC, Canada; Computar TEC55 55 mm telecentric lens; Computar, Cary, NC, USA) following our established procedures (30, 43). The consensual pupil responses were measured using 5,000 and 1,000 ms incremental pulses of five photoreceptor excitation combinations: [1] melanopsin-directed stimuli (17% Weber contrast) with no change in the excitation of the rods and three cone types, [2] L– and M–cones modulated in-phase to produce a cone luminance increment (+L+M; 10% Weber contrast) with no change in the excitation of S–cones, rods or melanopsin, [3] S–cone increments (+S; 10% Weber contrast) with no change in the excitation of melanopsin, rods, L– and M–cones, [4] a counterphase equiluminant L– and M–cone modulation (+L–M; 6% Weber contrast) with no change in L+M cone luminance or the excitation of S–cones, rods or melanopsin, and [5] the additive mixture of melanopsin (17% Weber contrast) with each of the photoreceptor combinations specified in [2–4].

The inter-stimulus interval included temporal white noise that randomly modulated the S–cone, M–cone, L–cone, and rod photoreceptor excitations (40% Michelson contrast) (44, 45) without changing the melanopsin photoreceptor excitation (10). The purpose of the temporal white noise is to limit the effect of any non-melanopsin photoreceptor absorptions on the melanopsin-directed pupil responses by desensitizing penumbral cones in the shadow of the retinal vasculature; for the 17% Weber contrast, melanopsin-directed pulse on the 2000 Td adaptation field, the penumbral L–, M–, and S–cone contrasts were 0.2, 0.5, and 0.6%, respectively and the rod contrast was 0.2%. The physically measured open-field cone contrast, which is the difference between the theoretical and measured irradiances of the five primary lights for the S M L R i photoreceptor excitations for the melanopsin-directed stimulus, was 0.0, 0.1, and 1.3% for the L–, M–, and S–cone contrasts, respectively, and 0.3% for the rod contrast. The rod contrast in all cone isolating conditions was ≤ 0.3%.

For the pupil measurements, each trial was separated by a 1 ms blank interval (46) and the trial repeated 10 times during a single recording sequence that was repeated at least 10 times (~100 trials per observer per stimulus condition; 8 conditions X 2 stimulus durations = ~1,600 total trials per observer). Testing sessions were <1.5 h to avoid the effect of observer fatigue and sleepiness on pupil responses. Data were measured during the day to minimize the influence of circadian variation on melanopsin-mediated pupil responses (9); each participant was scheduled at the same test time for their test sessions on different days.

### Analysis Metrics for the Pupil Light Responses

The PLR was quantified with reference to a baseline pupil diameter defined as the average of the 100 ms pre-stimulus data immediately before onset of the stimulus pulse. The PLR latency (in milliseconds) is the time to 1% pupil constriction after pulse onset; the peak constriction amplitude (% baseline diameter) is the smallest pupil diameter in response to stimulus onset, and the time at this maximum constriction is defined as the time to peak (in seconds). The pupil constriction velocity from stimulus pulse onset is the peak constriction amplitude divided by the time to peak (%.s^−1^). The light-adapted pupil diameter following stimulus offset (% baseline) was quantified at 1.8 s post-stimulus; although this metric is sometimes referred to as the post-stimulus pupil response (PSPR) when measured under light-adapted conditions (47), we adopted the more common notation, post-illumination pupil response (1.8 s PIPR). The pupil traces represent the global average of all repeats from all observers (~100 trials per observer per condition); the ±95% confidence limits were estimated from all stimulus trials for all observers for the respective stimulus conditions.

### Confirmation of Photoreceptor Isolation

We performed multiple control measurements to confirm the observer calibration and photoreceptor isolation. Firstly, a 500 ms, 18% Weber contrast rod incremental pulse with no change in melanopsin or cone excitations at a 5 Troland adaptation level was invisible after photopigment bleach and highly conspicuous after dark adaptation. Secondly, the cone excitations perceptually matching a 500 ms, 18% contrast rod incremental pedestal at a 5 Td background were equivalent to a decrease in L/[L+M], an increase in S/[L+M] and an increase in [L+M] (48). Finally, a 500 ms rod incremental pulse was invisible when presented at the maximum achievable contrast (18.5%) at a 5000 photopic Td adaptation level. The data clearly show that different photoreceptor-directed conditions produce pupil responses with different amplitude and timings. Individual differences in luminous efficiency, including any effect from photoreceptor polymorphisms, were corrected for during the HFP, as were individual differences in lens density (6, 10).

## Results

**Figure 1:**
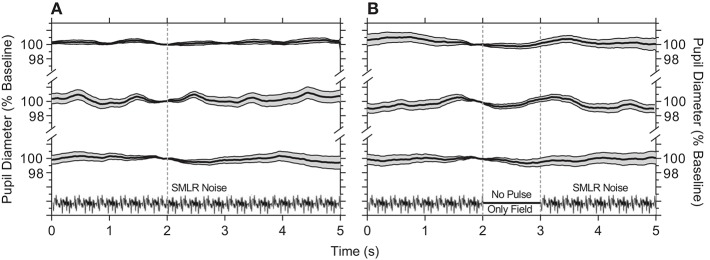
Pupil responses to temporal white noise. **(A)** Temporal white noise is presented for 5,000 ms then repeated; pupil diameter is steady during continuous presentation of the temporal white noise. **(B)** A 1,000 ms blank equal to the time average chromaticity and retinal illuminance of the orange field (no pulse, only field) is inserted within the temporal white noise; this blank field does not cause a pupil constriction. Panels show the average ±95% confidence limits for each of three observers (traces vertically offset; ~100 trials per observer). Pupil responses are normalized to the diameter at 2 s (vertical line) during each 5,000 ms repeat.

We first established that continuous presentation of the temporal white noise (i.e., no stimulus) that randomly modulates the S–cone, M–cone, L–cone, and rod photoreceptor excitations (without changing the melanopsin photoreceptor excitation) does not produce a pupil constriction (Figure 1A). The hippus evident in the pupil traces may be due to parasympathetic nervous system activity (49). Similarly, turning the noise off for a period equal to a 1,000 ms stimulus pulse and during which time this blank is equal to the time average illuminance of the adapting field, there is also no change in the pupil response (Figure 1B).

**Figure 2:**
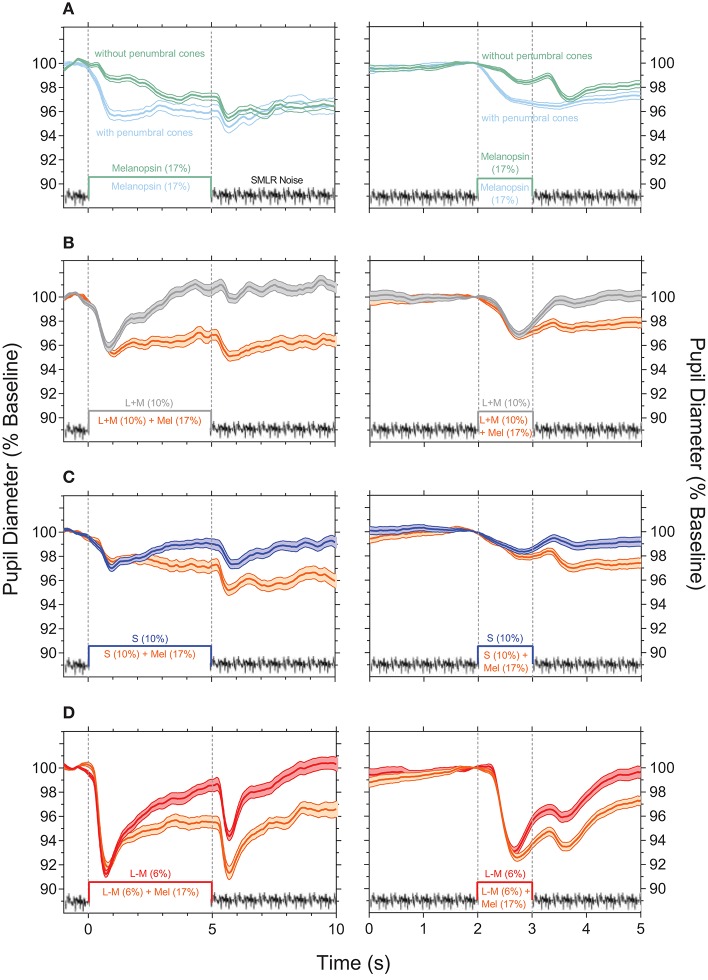
Light-adapted pupil responses measured under photoreceptor isolating conditions and with combined cone- and melanopsin-directed stimuli. **(A)** Melanopsin-directed pupil responses (17% Weber contrast in all measurements) measured with temporal white noise (without penumbral cones; green lines) and without temporal white noise (with penumbral cones; cyan lines). **(B)** +L+M cone luminance directed pupil responses (10% Weber contrast; grey lines) and the combined+ L+M cones and melanopsin responses (orange lines). **(C)** S-cone directed pupil responses (10% Weber contrast; blue lines) and the combined S-cone and melanopsin responses (orange lines). **(D)** +L–M directed pupil responses (6% Weber contrast; red lines) and the combined +L–M and melanopsin responses (orange lines). In all panels the data show the average ±95% confidence limits of 4 observers (~100 trials per observer). Dotted vertical lines indicate the onset and offset of the incremental pulses. Left column shows the PLR with 5,000 ms incremental pulses; right column shows the PLR with 1,000 ms incremental pulses. The average light-adapted baseline pupil diameter for all observers across all conditions was 4.43 mm ± 0.21 (mean ± SEM).

Pupil responses to melanopsin-directed stimuli measured with and without temporal white noise reveal the independent contribution of melanopsin (Figure 2A); the pupil responses for the 5,000 ms pulses are shown in the left panels, and for the 1,000 ms pulses in the right panels. The transient pupil constriction to the onset of a melanopsin-directed stimulus pulse is generated by penumbral cone signals (Figure 2A, cyan line); desensitizing penumbral cones and any residual high and low frequency cone responses using the temporal white noise (10) uncovers the signature melanopsin pupil response which includes a latency to constriction (5,000 ms pulse: 633.3 ± 43.3 ms; 1,000 ms pulse: 612.5 ± 42.7 ms) that is longer than for cones, with a slower velocity to constriction (5,000 ms pulse: 0.8 ± 0.02 %.s^−1^; 1,000 ms pulse: 2.3 ± 0.4 %.s^−1^) that remains sustained post-stimulus offset (Figure 2A, green lines; Table 1). For melanopsin-directed stimuli, the pupil responses to 5,000 ms pulses have a slower velocity to constriction than to 1,000 ms pulses (Table 1) because the velocity of the sustained melanopsin-mediated pupil constriction during light stimulation decreases over time. The time to peak constriction is 4.1x slower than for the average cone-directed PLR (5,000 ms; Table 1).

**Table 1:** The pupil light reflex (PLR) metrics (mean ± SEM) with 5,000 ms pulses and 1,000 ms pulses for different photoreceptor isolating conditions.

	**Photoreceptor directed stimulation**						
**Pupil metrics** **5,000 ms pulse**	**Mel** (17%)^*^	**L+M (10%)**	**S** **(10%)**	**L–M (6%)**	**L+M** **+** **Mel**	**S** **+** **Mel**	**L–M** **+** **Mel**
PLR Latency (ms)	633.3 ± 43.3	341.7 ± 49.3	491.7 ± 64.4	325.0 ± 4.8	412.5 ± 45.8	579.2 ± 81.5	354.2 ± 12.5
Peak Constriction Amplitude (%)	3.2 ± 0.2	4.5 ± 0.6	3.1 ± 0.2	8.9 ± 0.4	4.8 ± 0.3	3.8 ± 0.4	8.5 ± 0.7
Time to Peak (s)	4.0 ± 0.3	0.9 ± 0.1	1.2 ± 0.2	0.8 ± 0.04	1.7 ± 0.6	2.8 ± 0.8	0.9 ± 0.1
Constriction Velocity (%.s^−1^)	0.8 ± 0.02	5.0 ± 1.0	2.6 ± 0.3	11.1 ± 1.0	3.6 ± 0.8	1.7 ± 0.4	9.8 ± 1.2
1.8 s PIPR (%)	3.9 ± 0.7	0.1 ± 0.1	2.1 ± 0.5	2.1 ± 1.0	3.9 ± 0.7	4.1 ± 0.6	5.4 ± 0.6
**Pupil metrics** **5,000 ms pulse**	**Mel** (17%)^*^	**L+M (10%)**	**S** **(10%)**	**L–M (6%)**	**L+M** **+** **Mel**	**S** **+** **Mel**	**L–M** **+** **Mel**
PLR Latency (ms)	612.5 ± 42.7	395.8 ± 46.3	445.8 ± 114.3	366.7 ± 9.6	379.2 ± 48.8	425.0 ± 62.9	341.7 ± 22.1
Peak Constriction Amplitude (%)	2.1 ± 0.2	3.1 ± 0.2	2.1 ± 0.3	7.0 ± 1.0	3.4 ± 0.2	2.5 ± 0.4	7.6 ± 0.7
Time to Peak (s)	0.9 ± 0.1	0.8 ± 0.03	0.9 ± 0.1	0.7 ± 0.01	0.9 ± 0.1	0.8 ± 0.1	0.8 ± 0.03
Constriction Velocity (%.s^−1^)	2.3 ± 0.4	4.1 ± 0.3	2.6 ± 0.6	9.9 ± 1.6	4.1 ± 0.5	3.0 ± 0.3	10.0 ± 1.3
1.8 s PIPR (%)	1.9 ± 0.1	0.3 ± 0.2	1.1 ± 0.3	0.6 ± 0.1	2.1 ± 0.1	2.4 ± 0.4	3.0 ± 0.3

*The units for the metrics and Weber contrasts of the photoreceptor isolating conditions are given in the parentheses. Mel, melanopsin; PIPR, post-illumination pupil response*.

* *Measured with temporal white noise (without penumbral cones)*.

The cone photoreceptor-initiated pupil responses (Figures 2B–D) include higher transience (+L–M > +L+M > S–cone) than for melanopsin, with faster constriction latencies (range for 1,000 and 5,000 ms pulses: 325 to 491 ms), higher velocities (range for 1,000 and 5,000 ms pulses: 2.6 to 11.1%.s^−1^), and larger peak amplitudes to light onset (Table 1). That the stimulus contrast was ~30x higher than the +L–M visual detection threshold and ~2x higher than the +L+M detection threshold (10) resulted in the +L–M directed stimuli producing larger constriction amplitudes and higher constriction velocities than did +L+M directed stimuli. For cone-directed pulses (no change in the melanopsin excitation), the pupil rapidly redilates to baseline after stimulus offset whereas melanopsin-directed pulses produce sustained post-stimulus constrictions (1.8 s PIPR range for 1,000 ms cone-directed pulses: 0.3 to 1.1% vs. 1.9% for melanopsin-directed pulses; 5,000 ms cone-directed pulses: 0.1 to 2.1% vs. 3.9% for melanopsin-directed pulses) (Figures 2B–D; Table 1). The redilation component in response to luminance (+L+M) directed stimuli (Figure 2B, gray lines) is faster than that for S–cone (Figure 2C, blue lines) and +L–M directed stimuli (Figure 2D, red lines) and all show a second constriction between 291 and 425 ms after stimulus offset. We note that the observers verbally reported the presence of a prominent afterimage following offset of the +L–M and S–cone stimuli, and a faint afterimage following offset of the +L+M stimuli. When melanopsin combines with cone signals (Figures 2B–D, orange lines), the faster temporal response of cones mediates the transient pupil constrictions to stimulus onset and the slower melanopsin signal maintains the pupil constriction during continuous light stimulation and after stimulus offset, with a larger amplitude sustained constriction during the longer (5,000 ms) stimulus exposure. Together, these interactions reveal melanopsin- and cone-directed pupil responses at photopic illuminations under light-adapted conditions that provide no change in rod photoreceptor contrast.

**Figure 3:**
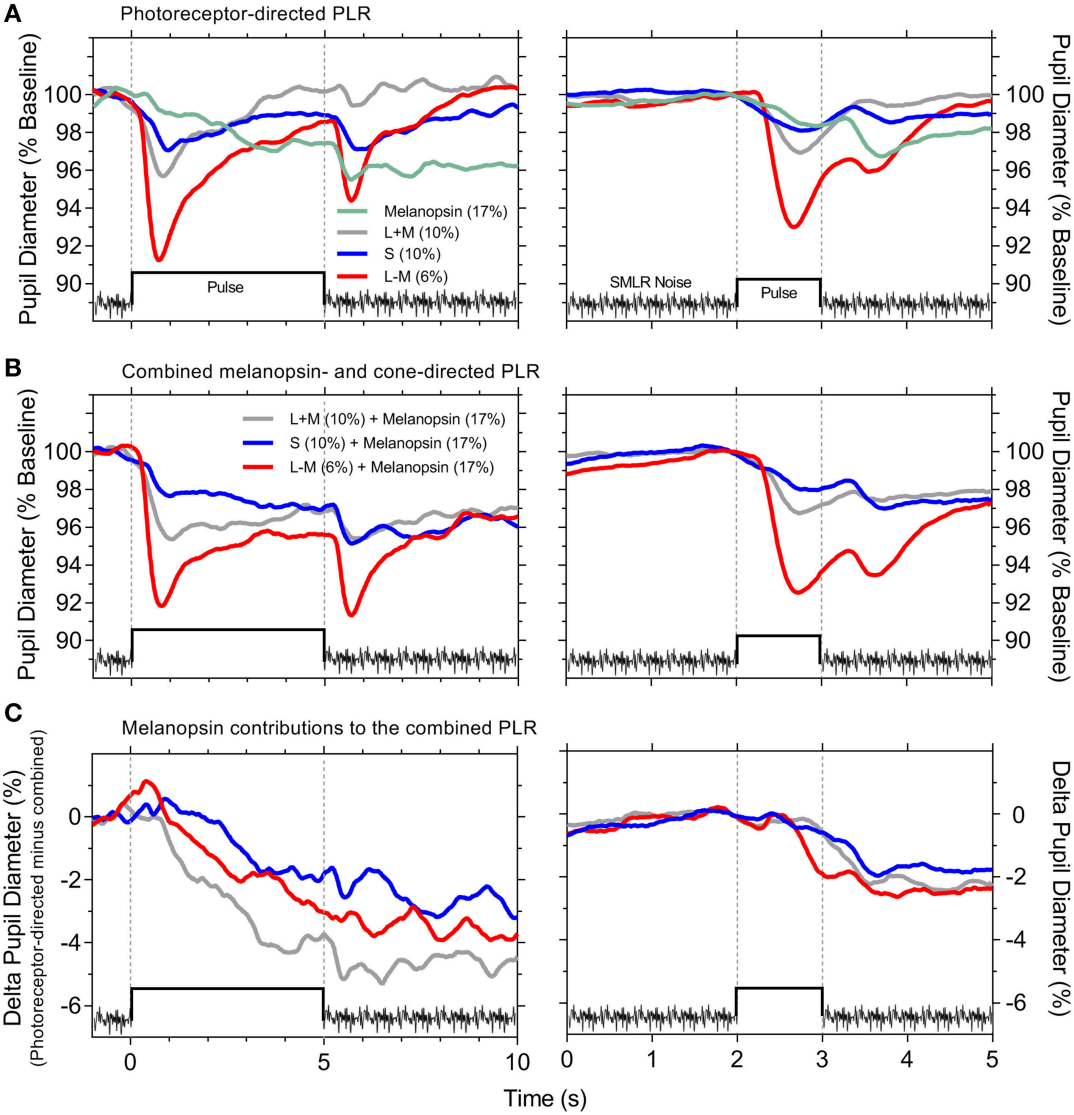
Photoreceptor-directed and combined pupil light responses (PLR). **(A)** Photoreceptor-directed PLR. **(B)** Combined melanopsin- and cone-directed PLR. **(C)** Melanopsin contributions to the combined PLR [difference between the data in **(A,B)**]. The PLR traces are an overlay of the average pupil responses from Figure 2 (*n* = 4 observers) on the same timescale for the 5,000 ms stimulus pulse (left panels) and 1,000 ms stimulus pulse (right panels). Stimulus contrasts are specified within the panels.

Overlaying all the photoreceptor-directed pupil light responses highlights the transient constriction generated by the cone signals, and the slower, sustained response generated by melanopsin (Figure 3A). The combined melanopsin- and cone-directed pupil responses (Figure 3B) show an initial transient constriction followed by a sustained constriction that is absent from the cone-directed pupil responses; the secondary constriction after stimulus offset is present in all conditions (Figures 3A,B). In Figure 3C, the difference between the photoreceptor-directed (Figure 3A) and the combined pupil responses (Figure 3B) reveals that melanopsin contributions to each of the cone-directed pupil responses manifests as a slow constriction to stimulus onset that remains sustained following stimulus offset, and which is equivalent to the melanopsin-directed pupil response (without penumbral cone intrusion; Figure 2A). For the combined pupil responses, the melanopsin contribution appears to be additive to the cone-directed inputs, with similar patterns for both the longer (5,000 ms) and shorter (1,000 ms) duration pulses.

## Discussion

We observe that the pupil light response is modulated by interactions between all three cone photoreceptor signals and melanopsin, with clear differences in their relative contributions. The constriction response mediated intrinsically via melanopsin includes a longer latency and slower velocity than for cones (Figures 2A, 3A); the melanopsin-mediated sustained pupil constriction continues post-stimulus (Figures 2A, 3C). Importantly, this shows that under light-adapted conditions, the putative melanopsin contribution to the pupil after stimulus offset (i.e., post-stimulus) mirrors the sustained post-illumination pupil response observed in the dark. Therefore, irrespective of adaptation condition, the implication is that the sustained activity of inner retinal melanopsin-expressing ipRGCs in response to the lighting conditions (i.e., stimulus and/or mean adaptation level) will set the light-adapted pupil diameter, as it does after stimulus offset in the dark, analogs to the post-illumination pupil response (2, 30). In comparison, cone-mediated pupil responses to changes in image contrast are transient with a rapid redilation to the light-adapted baseline pupil diameter.

For cone isolated tonic pupil responses (i.e., no change in melanopsin excitation), the S–cone directed stimuli (~1.4x visual threshold) produce a robust second pupil constriction at stimulus offset that is, relative to the respective peak pupil constriction, larger than for the chromatic +L–M stimuli (~30x visual threshold and which produce color opponent after-images) and luminance +L+M stimuli (~2x visual threshold) (Figure 3A and Table 1); these findings indicate that pupil responses to S–cone directed incremental lights (27, 28) reveal inhibitory inputs to the pupil pathway, as observed for phasic pupil responses to periodic modulation (6, 8, 10, 12). Such inhibitory responses are also present with the chromatic +L–M directed incremental pulses, that with flicker stimuli may indicate antagonism between the opponent cone inputs (50, 51). Residual-cone input is not likely to drive this second constriction in the melanopsin-directed pupil responses because the noise does not produce a transient pupil constriction (Figure 1). Recordings from ipRGCs in primate retina do however, reveal a transient hyperpolarization at light offset (2) and so the secondary constriction may therefore originate in ipRGCs, as the major pathway of outer retinal signals to the OPN. Another possibility is that this secondary constriction is related to the colored afterimage (28, 52, 53). Illusory changes in brightness can also induce a pupil constriction (54). That this secondary constriction is more prominent with both the longer duration (5,000 ms) melanopsin-directed pulses (with penumbral cone intrusion) and the cone-directed pulses (Figure 3A; left vs. right panels), indicates that temporal adaptation differentially alters the strength of afterimage (consistent with the observer reports) and therefore the amplitude of the second constriction.

As for mice, stimulus duration is an important determinant of the photoreceptor inputs to the afferent pupil response in humans. Transgenic mouse models however, show weak cone contributions to the pupil; transient pupil responses in mice are driven predominantly through the relay of rod signals to ipRGCs, through persistent, sustained pupil responses from ipRGCs during continuous light stimulation (22) and additive cone and melanopsin inputs that contribute to constriction (55). Here we show that cone signals drive human tonic pupil responses (Figure 1), in addition to rods (5, 7, 25). With melanopsin-directed stimuli, the latency to constriction is 292 ms longer than to a +L+M–cone luminance directed stimulus (5,000 ms pulse; Table 1 and Figure 3), strikingly similar to the ~280 ms difference in constriction latency between melanopsin only (rod-cone knockout) and wild-type mice (17). Such similarities serve to highlight the precision of the silent-substitution methodology for isolating melanopsin-mediated photoreceptor responses.

For the tonic pupil constriction to narrowband, aperiodic pulsed stimuli, the primary view is that the most sensitive outer or inner retinal process will mediate the constriction (i.e., winner take all) (25); stimulus irradiances that are suprathreshold for a melanopsin photoresponse increase channel membrane openings and decrease input impedance to shunt outer retinal signals extrinsically to ipRGCs (25). This study shows that when illumination conditions drive both, melanopsin and cones, the tonic pupil constrictions are always dominated by cones because of the slower constriction velocity of melanopsin, whereas during prolonged light exposure, melanopsin combines with cones to maintain constriction, then after stimulus offset the light-adapted pupil diameter is controlled by melanopsin.

## Ethics Statement

All experimental protocols were approved by the Queensland University of Technology (QUT) Human Research Ethics Committee (approval no: 1700000510) and conducted in accordance with their guidelines. Test protocols were completed in compliance with the tenets of the Declaration of Helsinki and all participants provided informed and written consent after the nature and possible consequences of the experiments were explained.

## Author Contributions

AZ, BF, and DC conceived, designed, and supervised the project. PA and AZ performed the experiments. All Authors participated in the analysis and interpretation of the experiments. AZ and BF wrote the manuscript. All Authors critically revised and approved the final manuscript version.

### Conflict of Interest Statement

The authors declare that the research was conducted in the absence of any commercial or financial relationships that could be construed as a potential conflict of interest.
